# Detecting transposable elements in long-read genomes using sTELLeR

**DOI:** 10.1093/bioinformatics/btae686

**Published:** 2024-11-18

**Authors:** Kristine Bilgrav Saether, Jesper Eisfeldt

**Affiliations:** Department of Molecular Medicine and Surgery, Karolinska Institute, Stockholm 171 76, Sweden; Clinical Genomics Facility, Science for Life Laboratory, Stockholm 171 76, Sweden; Department of Molecular Medicine and Surgery, Karolinska Institute, Stockholm 171 76, Sweden; Clinical Genomics Facility, Science for Life Laboratory, Stockholm 171 76, Sweden; Department of Clinical Genetics and Genomics, Karolinska University Hospital, Stockholm 171 77, Sweden

## Abstract

**Motivation:**

Repeat elements, such as transposable elements (TE), are highly repetitive DNA sequences that compose around 50% of the genome. TEs such as *Alu*, SVA, HERV, and L1 elements can cause disease through disrupting genes, causing frameshift mutations or altering splicing patters. These are elements challenging to characterize using short-read genome sequencing, due to its read length and TEs repetitive nature. Long-read genome sequencing (lrGS) enables bridging of TEs, allowing increased resolution across repetitive DNA sequences. lrGS therefore present an opportunity for improved TE detection and analysis not only from a research perspective but also for future clinical detection. When choosing an lrGS TE caller, parameters such as runtime, CPU hours, sensitivity, precision, and compatibility with inclusion into pipelines are crucial for efficient detection.

**Results:**

We therefore developed sTELLeR, (s) Transposable ELement in Long (e) Read, for accurate, fast, and effective TE detection. Particularly, sTELLeR exhibit higher precision and sensitivity for calling of *Alu* elements than similar tools. The caller is 5–48× as fast and uses <2% of the CPU hours compared to competitive callers. The caller is haplotype aware and output results in a variant call format (VCF) file, enabling compatibility with other variant callers and downstream analysis.

**Availability and implementation:**

sTELLeR is a python-based tool and is available at https://github.com/kristinebilgrav/sTELLeR. Altogether, we show that sTELLeR is a fast, sensitive, and precise caller for detection of TE elements, and can easily be implemented into variant calling workflows.

## 1 Introduction

Transposable elements (TEs) are repetitive genomic sequences capable of changing their genomic location. There are two subtypes of TEs, DNA transposons and retrotransposons (RTs). DNA transposons move through a cut-and-paste mechanism, make up around 2% of the genome (Chenais 2022) and are not active in human genomes (Solyom and Kazazian 2012). RTs change their location through a copy–paste mechanism involving an RNA intermediate and make up around 50% of the genome. There are different families of RTs, where the most common ones are elements L1, *Alu*, SVA, and HERV. Some of these remain active, and the transposition rates for *Alu*s range from 1:29–40 births to 1:63–117 births for L1 (Feusier *et al.* 2019, Borges-Monroy *et al.* 2021).

There are several examples where TEs have been disease causing, such as an SVA causing exon-trapping in *MFSD8* (MIM# 610951) (Kim *et al.* 2019), and *Alu* insertions disrupting exons in *NF1* and *USH2A* (Bilgrav Saether *et al.* 2023). Additionally, HERVs have been connected to cancer as well as autoimmunity (Alcazer *et al.* 2020). Detection of TEs is therefore clinically important, and understanding their mechanisms and characteristics is useful for determining their genomic consequences.

Long-read whole genome sequencing (lrGS) enables base-pair resolution of >10 kb stretches of continuous DNA. This facilitates characterization and resolution of complex and dynamic genomic regions (Logsdon *et al.* 2020). lrGS enabling full-length base-pair resolution of TEs is a significant improvement from short-read genome sequencing (srGS) where read lengths of 150 bp limit the discovery of genomic variation across repeat regions such as tandem repeats, segmental duplications, and TEs (Logsdon *et al.* 2020). Due to the previous complexity of analyzing these regions, they remain largely understudied.

Although the reference genome contains plenty of TEs, the majority of them differ across the population and are not represented in the reference genome (Ewing and Kazazian 2010, Sudmant *et al.* 2015, Bilgrav Saether *et al.* 2023). We have previously implemented an srGS nonreference TE insertion (TEI) detection workflow into our clinical analysis pipeline (Bilgrav Saether *et al.* 2023). However, as previously discussed, srGS provides limited resolution of TEs. With lrGS becoming more accessible and clinically applicable, an accurate, robust, and time-efficient lrGS TE caller is necessary in order to identify TEI. We here present a novel (*s*) *T*ransposable *EL*ement *L*ong (*e*) *R*ead (sTELLeR) caller, which is fast, sensitive, and precise at identifying nonreference TEI. We apply sTELLeR on simulated data as well as on a genome in a bottle (GIAB) trio and samples from the Human Pangenome Reference Consortium (HPRC).

## 2 Materials and methods

### 2.1 sTELLeR algorithm

**Figure 1.**
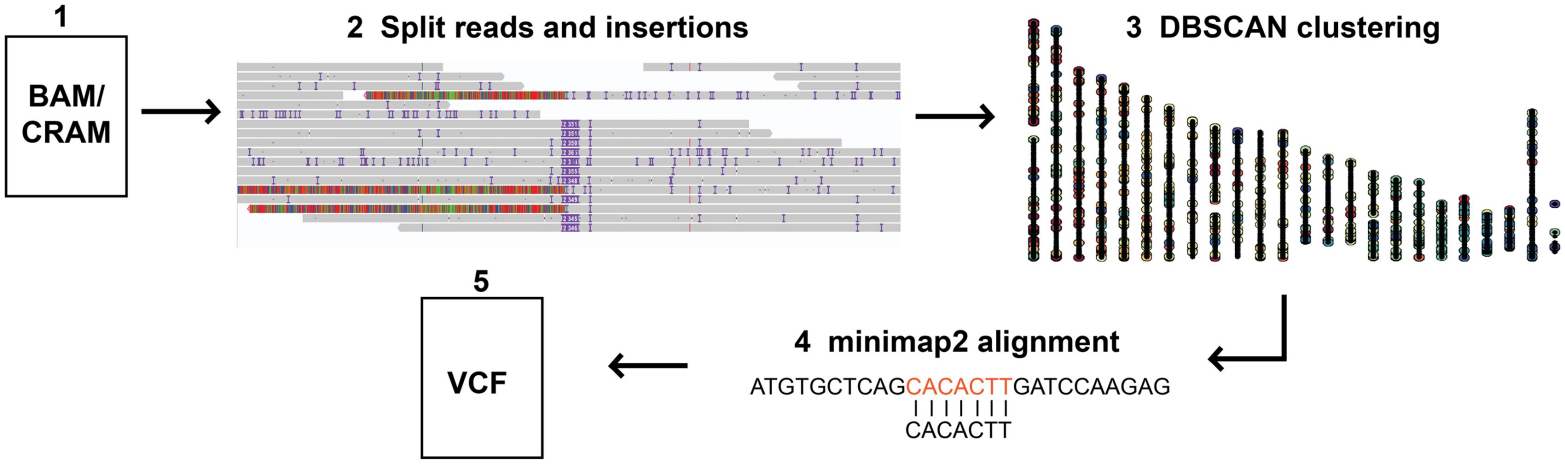
sTELLeR algorithm overview. (1) BAM or CRAM file can be provided as input. (2) sTELLeR identifies split reads and insertions. (3) The split reads and insertions are clustered using DBSCAN. (4) Sequences of clustered split reads and insertions are aligned to TE sequences provided. (5) Resulting matches are provided in a variant call format (VCF) file. Image adapted from (https://github.com/kristinebilgrav/sTELLeR).

The sTELLeR algorithm entails five steps (Fig. 1). sTELLeR takes a bam or a cram file as input (1) and (2) extracts positions of split reads and insertions. The positions are (3) clustered using the density-based spatial clustering of applications with noise (DBSCAN) clustering algorithm. DBSCAN will cluster positions based on proximity to each other, where *ε* determines maximum distance between neighbors and a minimum number of positions is set to consider it a cluster. Once clusters are obtained, (4) the sequence of the insertions and split reads spanning the cluster is extracted and aligned to TEs which are provided in a fasta file using minimap2. Finally, (5) the aligned sequences are refined to a consensus nucleotide position and filtered to only include clusters where a minimum number of reads (user-defined, default 3) match a TE, and the length of the match needs to be at least 10% of the original insertion or split read. Lastly, results are provided as a VCF output.

### 2.2 sTELLeR benchmarking

sTELLeR was compared to multiple state-of-the-art lrGS TE callers, including xTEA, PALMER, TELR, and TLDR (Ewing *et al.* 2020, Shahid and Slotkin 2020, Chu *et al.* 2021, McDonald *et al.* 2021). The callers were compared based on sensitivity, precision, and runtime. Callers that did not complete within 48 h were excluded from the benchmark. Callers also need to be stable and compatible with inclusion into pipelines in, e.g. Snakemake (Mölder *et al.* 2021) or Nextflow (Di Tommaso *et al.* 2017). All analyses were run on the high-performance cluster Uppsala Multidisciplinary Center for Advanced Computational Science (UPPMAX). Chosen callers were ran on data obtained from GIAB and the HPRC (Zook *et al.* 2016, Liao *et al.* 2023) as well as on simulated data.

The simulated data were created by inserting TE sequences from *Alu*, L1, HERV, and SVA at random in a masked reference file (GCF_000001405.26) (Supplementary File S1). The *Alu*, L1, and SVA sequences were obtained by extracting TE sequences from a GRCh38 reference file (GCF_000001405.26) using positions indicated by RepeatMasker. The HERV sequence was obtained from NCBI (AF020092) (Sayers *et al.* 2022). The exact positions were determined using the random positions. The fasta file containing TEs was used to create a simulated dataset using PBSIM3 (Ono *et al.* 2022). This resulted in a simulated BAM file with 20× coverage and where the reads have a similar error rate to those generated using the PacBio RS II. There were a total of 886 *Alu*, 888 L1, 444 SVA, and 452 HERV insertions in the dataset. Commands and scripts are given in Supplementary File S1.

An additional assembly-based callset was generated using the HPRC *de novo* assembly of the samples HG002 and HG01071. SVIM-asm (Heller and Vingron 2021) was utilized to call insertions on the assembly, and RepeatMasker (Smit *et al.* 2013) was used to determine the presence of *Alu*, L1, HERV, and SVA elements. Elements with a percent divergence >15% and under a certain consecutive length (SVA: 300, HERV: 1500, L1: 200, *Alu*: 100) were excluded. This resulted in 2193 *Alu*, 492 L1, 14 HERV, and 205 SVA elements in HG002 and 2262 *Alu*, 439 L1, 11 HERV, and 202 SVA elements in HG01071. sTELLeR, TLDR, and xTEA were subsequently ran on the PacBio samples downloaded from the same source and realigned to the GRCh38 reference (GCF_000001405.26) (Supplementary File S1).

For comparison of runtimes, PacBio BAM files for GIAB samples HG002, HG003, and HG004 were obtained from GIAB (Zook *et al.* 2016) and converted back to fastq files for alignment. ONT fastq files for the same samples were obtained from GIAB (Zook *et al.* 2016, Shafin *et al.* 2020). All fastq files were aligned to reference genome GRCh38 using minimap2 (Li 2018).

Commands for running the tools are listed in Supplementary File S1. The TE fasta sequences, used to run TLDR and sTELLeR, were downloaded from NCBI (Sayers *et al.* 2022) or extracted from the reference genome. These sequences are available through https://github.com/kristinebilgrav/sTELLeR_supplementary/, along with the scripts used to generate the assembly-based and simulated callset.

### 2.3 TE analysis in srGS

HG004 srGS bam file was downloaded from GIAB (Zook *et al.* 2016) and realigned to GRCh38 using bwa-mem (Li and Durbin 2009) and downsampled to 30× coverage. TE calling in the srGS data was performed using RetroSeq (Keane *et al.* 2013) and MELT2 (Gardner *et al.* 2017). The srGS TE caller RetroSeq is a caller shown to have high sensitivity in previous studies (Keane *et al.* 2013, Rishishwar *et al.* 2017, Vendrell-Mir *et al.* 2019). MELT2 is a popular TE caller which has been utilized to generate the GnomAD SV callset (Gardner *et al.* 2017, Collins *et al.* 2020). The commands used are given in Supplementary File S1.

## 3 Results

We downloaded and tested multiple callers such as xTEA (Chu *et al.* 2021), PALMER (McDonald *et al.* 2021), TELR (Han *et al.* 2022), and TLDR (Ewing *et al.* 2020). However, due to many of our mentioned demands from a caller, some fail to meet our requirements. PALMER was tested, but its runtime exceeded our runtime limits where it could not identify TEs on GS within 48 h. TELR (v1.1) was easy to install; however, a bug known to the developer, but not resolved, rendered it to be unstable on our data. A comprehensive table of runtimes for the excluded callers is found in Supplementary Table S1. The callers tested which were competitive to sTELLeR were TLDR and xTEA.

Runtime, CPU hours, and memory usage of sTELLeR, TLDR, and xTEA were assessed by running the tools on the GIAB Ashkenazim trio using the PacBio and ONT data (Table 1). Runtimes were faster for sTELLeR across all sample types and techniques.

**Table 1.** sTELLeR, TLDR, and xTEA runtimes and memory usage for GIAB samples.

Dataset	Coverage	Caller	Walltime (min)	CPU time (walltime×CPU)	Memory (GB)
HG002 PacBio	52×	sTELLeR	21	21	6
		TLDR	111	1110	23
		xTEA	1020	8161	12
HG002 ONT	43×	sTELLeR	83	83	2
		TLDR	231	2310	69
		xTEA	2136	17 083	12
HG003 PacBio	61×	sTELLeR	27	27	8
		TLDR	127	1265	25
		xTEA	1112	8893	13
HG003 ONT	78×	sTELLeR	149	149	3
		TLDR	468	7481	105
		xTEA	2328	18 621	12
HG004 PacBio	60×	sTELLeR	22	22	14
		TLDR	135	1346	20
		xTEA	1108	8866	12
HG004 ONT	78×	sTELLeR	160	160	3
		TLDR	471	7537	100
		xTEA	2435	19 480	14

sTELLeR, TLDR, and xTEA were further tested on simulated data produced and run as described in Section 2. The simulated dataset contained 886 *Alu*, 888 L1, 452 HERV, and 444 SVA insertions. sTELLeR was able to identify 863 *Alu*, 859 L1, 443 HERV, and 435 SVA elements; TLDR 883 *Alu*, 52 L1, 186 HERV, and 14 SVA elements; and xTEA 866 *Alu*, no L1 or HERV, and 430 SVA elements (Table 2). This results in a sensitivity of 0.97 for *Alu* detection with sTELLeR, 0.99 using TLDR and 0.97 using xTEA. The precision was 1 for both sTELLeR and xTEA, and 0.97 for TLDR (Table 2). For L1 detection, the sensitivity was 0.96 for sTELLeR and 0.05 for TLDR and the precision 1 for sTELLeR and 0.82 for TLDR. For HERV, sTELLeR had a sensitivity of 0.98 and a precision of 1, while TLDR 0.41 and 1, respectively. For SVA, sTELLeR had a precision of 1, xTEA 0.99, while TLDR 0.82. The sensitivity was 0.97 for sTELLeR, 0.98 for xTEA, and 0.03 for TLDR.

**Table 2.** Simulated metrics for the callers sTELLeR, TLDR, and xTEA ran on a simulated dataset (*Alu* = 886, L1 = 888, HERV = 452, SVA = 444).

		sTELLeR	TLDR	xTEA
True positives	*Alu*	863	883	866
	L1	859	52	NA
	HERV	443	186	NA
	SVA	435	14	429
Sensitivity	*Alu*	0.97	0.99	0.97
	L1	0.96	0.05	NA
	HERV	0.98	0.41	NA
	SVA	0.97	0.03	0.98
Precision	*Alu*	1.0	0.97	1.0
	L1	1.0	0.82	NA
	HERV	1.0	1.0	NA
	SVA	1.0	0.82	0.99

NA: Not applicable.

Furthermore, the callers were run on PacBio data from samples HG002 and HG01071. Resulting TEs were compared to SVIM-asm (Heller and Vingron 2021) insertion calls determined to be *Alu*, L1, HERV, or SVA elements by RepeatMasker (Smit *et al.* 2013). For HG002 (TEI = 2904), sTELLeR was able to call 1885 true positive TEI, TLDR 1741, and xTEA 1543. For HG01071 (TEI = 2914), sTELLeR identified 1914, TLDR 1808, and xTEA 1645 true positive TEI (Table 3).

**Table 3.** Metrics for the callers sTELLeR, TLDR, and xTEA on samples HG002 (*Alu* = 2193, L1 = 492, HERV = 14, SVA = 205) and HG01071 (*Alu* = 2262, L1 = 439, HERV = 11, SVA = 202).

		sTELLeR		TLDR		xTEA	
		HG002	HG01071	HG002	HG01071	HG002	HG01071
True positives	*Alu*	1572	1592	1494	1556	1357	1408
	L1	213	232	152	164	186	186
	HERV	4	5	4	5	0	0
	SVA	96	85	91	83	0	51
Sensitivity	*Alu*	0.71	0.70	0.68	0.68	0.61	0.62
	L1	0.43	0.52	0.30	0.37	0.37	0.33
	HERV	0.28	0.45	0.03	0.45	0	0
	SVA	0.46	0.42	0.44	0.81	0	0.02
Precision	*Alu*	0.87	0.92	0.86	0.88	0.86	0.87
	L1	0.54	0.36	0.63	0.61	0.84	0.73
	HERV	0.36	0.17	0.05	0.07	0	0
	SVA	0.75	0.77	0.45	0.94	0	0.94

**Figure 2.**
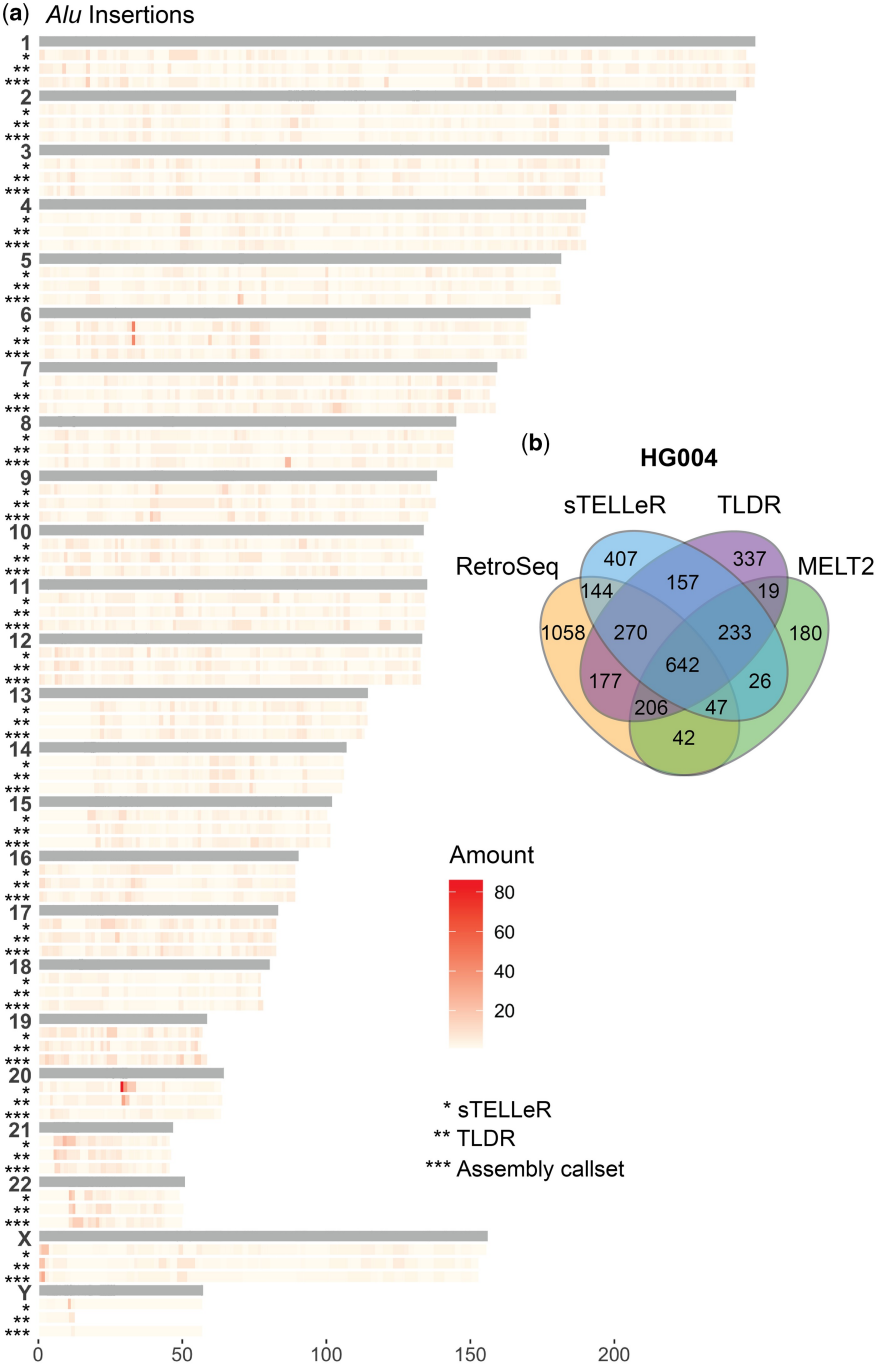
sTELLeR applied. (a) Heatmap of binned *Alu* insertions identified across HG002 and HG01071 with sTELLeR, TLDR, and the assembly-based callset. (b) VENN diagram of overlapping TEs called by RetroSeq, MELT2, sTELLeR, and TLDR in HG004.

By binning the number of *Alu* elements across HG002 and HG01071 identified using the two most competitive callers (sTELLeR, TLDR) along with the assembly-based callset, one can observe clusters of *Alu* elements across the genome (Fig. 2a). The three methods have a similar distribution, where notably the acrocentric p-arms have low amount of *Alu* elements, while a region on the p-arm of X has high amounts. sTELLeR and TLDR share similar patterns of *Alu* detection and with a few exceptions is similar to the assembly-based callset. To further assess sTELLeR and TLDR, along with TE detection in lrGS, we called TEs *Alu* and L1 in sample HG004 using both srGS and lrGS. We ran sTELLeR and TLDR on the PacBio lrGS data and RetroSeq and MELT2 on the srGS data (Fig. 2b). We found the overlap of all callers to be 642. The total number of TEs undetected by the lrGS callers were 1280, while sTELLeR and TLDR agreed upon 1302 TEI.

## 4 Discussion

lrGS has come forth as a technique valuable for bridging repeats and regions previously challenging to resolve (Shahid and Slotkin 2020, Bilgrav Saether *et al.* 2023). Analysis of TEs have previously suffered from short-read lengths in srGS, as the length of repeats can be longer than a read. Thus, lrGS open for in-depth studies and characterization of TEs within the genome which was previously challenging. TEs implication in disease (Solyom and Kazazian 2012, Chenais 2022) makes them an important candidate in genome analysis, which have been underrepresented in clinical analysis workflows (Bilgrav Saether *et al.* 2023). In lrGS, a nonreference TE will be represented as insertions and split reads when aligned to a reference genome. The split reads or insertions will contain the sequence of the inserted element. This also applies to most other larger structural variants (SVs), and it is therefore necessary to be able to differentiate between TEIs and other SVs. With this, we wanted a TE caller for application on lrGS which can assist in both characterization and research of TEs as well as being compatible with future implementation of lrGS analysis workflows.

Implementation into research as well as diagnostic workflows require a fast, sensitive, and reproducible TE caller. A program should be easy to install and run, as well as be compatible with running in a pipeline environment. It should not be too computationally demanding. Ideally it should output a VCF in order to be compatible with outputs from other variant callers such as single-nucleotide variant, SV and copy-number variant callers. Many available callers today are often collected in a pipeline format, demanding extensive configuration. This makes further addition into in-house pipelines a complicated and inefficient step. Additionally, some callers explored had compilation errors or a runtime exceeding >48 h, which we deem too long for our analysis. The tested callers fit for our use were TLDR and xTEA. TLDR has been shown to achieve a sensitivity similar to srGS TE callers while assembling and annotating the insertions. However, the caller output results in a table format, and is not haplotype aware. xTEA display high sensitivity for *Alu* detection in both PacBio and ONT data (Chu *et al.* 2021); however, runtimes range from 22 to 46 h and output results in a text file format. Thus, in order to fulfill our requirements, we developed sTELLeR.sTELLeR is a python-based caller designed to identify nonreference insertions across the genome. Detection of reference TE polymorphisms, i.e. TEs present in the reference, but missing from the individuals, is not possible. The sTELLeR algorithm involves detecting split reads and insertions, upon which their positions are clustered using DBSCAN. DBSCAN clusters positions based on their distance to each other. The distance and number of positions needed to form a cluster can be altered (–sr) and optimized for intended use. This enables the sensitivity adaptable, providing flexibility and tailoring of the caller to the user’s needs. The user-input fasta file of TEs allows flexibility, making it possible to detect any type of nonreference insertion. sTELLeR is haplotype aware and can run on genome assemblies. Additionally, the output is provided in VCF output and any bam file can be submitted, meaning the tool can be applied to any species and nonreference insertion.

In research as well as clinical analysis, time, sensitivity, precision, and compatibility are important aspects. We show sTELLeR to be all the above, with easy installation through git or container, a runtime <30 min for a 60× PacBio genome (Table 2). Compared to similar tools such as TLDR and xTEA (Ewing *et al.* 2020, Chu *et al.* 2021), sTELLeR is 5–48× as fast and uses <2% of the CPU time.sTELLeR, TLDR, and xTEA were tested on a simulated dataset containing 886 *Alu*, 888 L1, 452 HERV, and 444 SVA insertions, as well as on samples HG002 and HG01071 from the HPRC (Wang *et al.* 2022). For the simulated dataset, sTELLeR has higher (TLDR) or similar (xTEA) precision and similar (xTEA) or slightly lower (TLDR) sensitivity for analysis of *Alu* elements than xTEA and TLDR (Table 2). For analysis of L1 elements, sTELLeR has higher sensitivity and precision than TLDR. Across both HERV and SVA elements, sTELLeR outperforms TLDR in both sensitivity and precision. xTEA is competitive at detecting SVA insertions. In the simulated dataset, xTEA was not able to identify any L1 or HERV elements, which could be due to the internal TE dataset not being compatible with the sequence of the elements used in the simulation, although retrieved from either the reference genome (GCF_000001405.26) or NCBI (AF020092).

In results from HG002 and HG01071, sTELLeR is more sensitive than both xTEA and TLDR for *Alu* and L1 element detection and more precise at *Alu* detection (Table 3). For SVA detection, sTELLeR has a precision >0.75 and sensitivity >0.42, while TLDRs sensitivity vary from 0.44 to 0.81 with a precision around 0.45. xTEA was not able to identify any SVA in sample HG01071 and for sample HG002 the sensitivity was very low (0.02), although the calls made were accurate (precision of 0.94). Furthermore, no HERV elements were detected by xTEA. HERV detection can be challenging as these elements are large, polymorphic and often not full length in the human population (Belshaw *et al.* 2005, Garcia-Montojo *et al.* 2018, Xue *et al.* 2020). This is reflected in the absence of true positive calls by xTEA and the low precision rate of both TLDR (<0.07) and sTELLeR (<0.36) on samples HG002 and HG01071 (Table 3). Overall, sTELLeR is able to identify a larger number of true positives across all TE types. Potential false positives can later be filtered using population databases. These results along with the low CPU usage show sTELLeR to be an efficient, precise, and sensitive TE caller.

In conclusion, we have developed a sensitive and precise caller which is fast and highly compatible with implementation into in-house workflows for research as well as clinical analysis.

## Supplementary Material

btae686_Supplementary_Data

## Data Availability

sTELLeR can be found at https://github.com/kristinebilgrav/sTELLeR. A container is available as a docker image at https://hub.docker.com/r/kristinebilgrav/steller. Scripts used to generate the assembly-based and simulated callset as well as fasta sequences other scripts used in the project can be found at https://github.com/kristinebilgrav/sTELLeR_supplementary/. HPRC samples can be found at https://github.com/human-pangenomics/HPP_Year1_Data_Freeze_v1.0. GIAB samples can be found at https://github.com/genome-in-a-bottle/giab_data_indexes.
