# Supplementary material for: Detecting transposable elements in long-read genomes using sTELLeR
Source: Bioinformatics. 2024 Nov 18;40(11):btae686. doi: 10.1093/bioinformatics/btae686 (PMC11601167; doi:10.1093/bioinformatics/btae686)
Supplement: btae686_Supplementary_Data [file btae686_supplementary_data.zip › sTELLeR_SupplementaryFile1.docx]

Supplementary File 1

TELLR benchmarking commands

**TE lrGS analysis**

Convert bam to fastq

samtools view -bh -u <input bam> | samtools sort -l 0 -m 6G -n -@ 16 - | samtools fastq - > $out.fastq

Alignment using minimap2

minimap2 -R '@RG\tID:'"$id"'\tSM:'"$id"'' -a -t 16 --MD -x map-<pb or ont> -y <reference> <fastq> | samtools view -Sbh - | samtools sort -m 4G -@16 - > $out.bam

samtools index $out.bam

Run xTEA

xtea_long -i <sampleid list file> -b <bam list file> -p <dir> --rep <replib> -r <ref> -y 3 --xtea <xtea executable> -f 31

bash sampleid/run_xTEA_pipeline.sh

Run PALMER

PALMER --input <bamfile> --workdir <path> --ref_ver GRCh38 --ref_fa <ref> --type ALU --mode raw --chr <chr> --output <outputid> --start 1 --end <end of chr>

Run sTELLeR

python steller/steller.py --ref <GRCh38 reference> --TE_fasta <tefasta> --bam <bam file> --sr 4 --style <pb or ont> -mr 80

Run TLDR

tldr -b < .bam file > -e <tefasta> -r <GRCh38 reference> -p 16 -m 4 -c <chromsomes.txt> --max_cluster 300

The chromosomes.txt file contained all autosomes and sex chromosomes. The TE fasta sequences for TLDR were the same as in sTELLeR. For xTEA we used the default TE reference provided alongside xTEA.

**TE srGS analysis**

Downsampling

samtools view -@6 -h -s 0.1 <bamfile> -bo $out.bam

samtools index $out.bam

BAM to fastq

samtools view -bh -u <bam> | samtools sort -l 0 -m 6G -n -@ 16 - | samtools fastq - > $out.fastq

Alignment

bwa mem -p -t 16 <reference> <fastq> | samtools view -Sbh - | samtools sort -m 3G -@ 16 - > $out.bam

samtools index $out.bam

Retroseq

retroseq.pl -discover -bam <bam> -output $out.discover.vcf -refTEs <reference bed w/TEs>

retroseq.pl -call -bam <bam> -input $out.discover.vcf -ref <reference> -output $out.called.R.vcf

MELT2

java -jar MELTv2.2.2/MELT.jar Single -bamfile <bam> -h <reference> -t <TE zip file list> -w <directory> -n <gene gff>

**Make simulated truthset:**

Insert TE sequences in a masked reference file:

import sys

import random

alu=<sequence>

herv=<sequence>

l1=<sequence>

sva=<sequence>

def findtepos(sequence, TEpos, TEpostotype):

length=len(sequence)

random_amount=random.randint(60,200) #how many TEs to insert

tecount=0

for i in range(0, random_amount): #insert TE random_amount of times

tecount += 1

if tecount == 1:

te=alu

testr='Alu'

elif tecount == 2:

te=l1

testr='L1'

elif tecount== 3:

te=herv

testr='HERV'

elif tecount== 4:

te=sva

testr='SVA'

elif tecount== 5:

te=alu

testr='Alu'

elif tecount== 6:

te=l1

testr='L1'

tecount=0

random_position=random.randint(0,length)

lst=[list(range(i-20000,i+20000)) for i in list(TEpos.keys())]

avoidlist=[item for sublist in lst for item in sublist]

if random_position not in avoidlist: #insert at random position in chr

TEpos[random_position]=te

TEpostotype[random_position]=testr

else:

continue

return TEpos, TEpostotype

def inserttes(sequence, TEpos): # dict with te pos (based on ori len) and which TE inserted

sortedtepos=sorted(list(TEpos.keys()), reverse=True)

for randpos in sortedtepos:

tetype=TEpos[randpos]

newsequence="{}{}{}".format(sequence[0:randpos],tetype,sequence[randpos:])

sequence=newsequence

return sequence

chrline={}

sequence=[]

readnames=[]

tetoreaddict={}

seqs={}

for line in open(sys.argv[1]): # Reference file, extrat reads for simulation

line = line.rstrip('\n')

if line.startswith('>'):

chr=line.split('>')[-1].split(' ')[0]

seqs[chr]=[]

else:

seqs[chr].append(line)

fastaoutput = open(sys.argv[2], 'w')

posoutput=open(sys.argv[3], 'w')

TEdict={}

TEtype={}

for s in seqs:

chr= s

print(chr)

TEdict[chr]={}

TEtype[chr]={}

seq=''.join(seqs[s]) #sequence

randompos=findtepos(seq, TEdict[chr], TEtype[chr]) #insert tes

newref=inserttes(seq, TEdict[chr])

teread=newref

header='>GRCh38_{}'.format(chr)

fastaoutput.write(header + '\n')

fastaoutput.write(teread + '\n')

sortedTEpos=sorted(list(TEdict[chr].keys()))

for p in sortedTEpos:

posoutput.write("\t".join([chr, str(p), TEtype[chr][p] ]) + '\n')

PBSIM3

Use fasta file generated above, containing TE insertions.

pbsim --strategy wgs --method qshmm --qshmm data/QSHMM-RSII.model --depth 20 --genome <fasta>

# cat all individual fastq files together

cat *fastq > pbsim.fastq

# Align to masked original reference

minimap2 -R "@RG\tID:<sampleID>\tSM:<sampleID>" -a -t 16 --MD -x map-pb -Y -y <reference> <fastq> | samtools view -Sbh - | samtools sort -m 4G -@16 - > <sampleID>.bam

call TEs using xTEA, TLDR and sTELLeR

**denovo assembly callset:**

# Commands used to generate a truthset from HPRC de novo assemblies HG002 and HG01071

# Align assembly

awk '/^S/{print ">"$2;print $3}' $1 > $2.fa

minimap2 -ax asm5 <reference> <fasta> | samtools view -Sbh - | samtools sort -m 4G -@16 - > <out.bam>

samtools index <out.bam>

# Run SVIM-asm

svim-asm diploid $home/ <hap1> <hap2> <reference> --sample <ID>

# Extract insertions from SVIM

grep -v '#' <SVIM.vcf> | grep INS | awk ' {print ">", $1,":", $2,":", $3, "\n", $5 }' > <INSfasta>

# Repeatmask insertions

RepeatMasker -pa 16 -qq -gff -species human <INSfasta> -dir .

# create bed file from RM output

grep <TE> <fasta.out> | awk 'OFS="\t" {print $5, $6, $7, $10, $11, $2}' > <SVIM.INS.Alu.out>
