# Supplementary material for: Detecting transposable elements in long-read genomes using sTELLeR
Source: Bioinformatics. 2024 Nov 18;40(11):btae686. doi: 10.1093/bioinformatics/btae686 (PMC11601167; doi:10.1093/bioinformatics/btae686)
Supplement: btae686_Supplementary_Data [file btae686_supplementary_data.zip › Supplementary_Table1.docx]

Supplementary Table 1: Excluded callers and their running parameters.

| Sample | Programming language | Caller | Walltime | CPU | Availability | Comment |
| --- | --- | --- | --- | --- | --- | --- |
| HG004 | C++ | PALMER | >48h | 16 | <https://github.com/WeichenZhou/PALMER> | Runtime exceeding 2 days |
| HG004 | Python | TELR | - | - | <https://github.com/bergmanlab/TELR> | Bug causing failure upon running |
